# Reduced IgM levels and elevated IgG levels against oxidized low-density lipoproteins in HIV-1 infection

**DOI:** 10.1186/1471-2334-14-143

**Published:** 2014-03-17

**Authors:** Aylin Yilmaz, Karin Jennbacken, Linda Fogelstrand

**Affiliations:** 1 Department of Infectious Diseases, Institute of Biomedicine, Sahlgrenska Academy, University of Gothenburg, Gothenburg, Sweden; 2 Wallenberg Laboratory, Institute of Medicine, Sahlgrenska Academy, University of Gothenburg, Gothenburg, Sweden; 3 Department of Clinical Chemistry and Transfusion Medicine, Institute of Biomedicine, all at Sahlgrenska Academy, University of Gothenburg, Gothenburg, Sweden

## Abstract

**Background:**

Chronic HIV infection is associated with increased risk of cardiovascular disease caused by atherosclerosis. Oxidized forms of low-density lipoprotein (LDL) are present in atherosclerotic lesions and constitute major epitopes for natural antibodies. IgM has been shown to be protective against atherosclerosis, whereas the role of corresponding IgG is less clear. The objective of this study was to determine if HIV + individuals have disturbed levels of IgM and IgG directed against oxidized forms of LDL as compared to HIV- individuals.

**Methods:**

Ninety-one HIV + patients and 92 HIV- controls were included in this retrospective study. Circulating levels of IgG and IgM directed against two forms of oxidized LDL; copper oxidized (OxLDL) and malondialdehyde modified (MDA-LDL), total IgM and IgG, C-reactive protein (CRP), soluble CD14, and apolipoproteins A1 and B were determined.

**Results:**

HIV + individuals had slightly lower levels of IgM against MDA-LDL and higher levels of IgG against MDA-LDL, OxLDL, and total IgG, than HIV- controls. Anti-MDA-LDL and Anti-OxLDL IgG displayed a positive correlation with viral load and a negative correlation with the CD4^+^ T-cell count. HIV + individuals also displayed elevated CRP and soluble CD14 levels compared to HIV- individuals, but there were no correlations between CRP or soluble CD14 and specific antibodies.

**Conclusions:**

HIV infection is associated with higher levels of IgG including specific IgG against oxidized forms of LDL, and lower IgM against the same epitope. In addition to dyslipidemia, immune activation, HIV-replication and an accumulation of risk factors for atherosclerosis, this adverse antibody profile may be of major importance for the increased risk of cardiovascular disease in HIV + individuals.

## Background

The use of antiretroviral treatment (ART) for HIV infection has led to a dramatic reduction of HIV-related morbidity and mortality, and the life expectancy of HIV-infected individuals is now approaching that of the general population [1-4]. As HIV-related mortality has decreased, there has been a relative increase in the proportion of deaths attributable to other complications such as renal disease, liver disease, neurocognitive impairment, and cardiovascular disease (CVD) [5]. For reasons that are not yet fully understood, HIV-infected individuals, even those on stable suppressive treatment, have a higher prevalence of atherosclerosis than age-matched HIV-negative adults [6-9]. This increased risk is independent of traditional risk factors for CVD such as high blood pressure, high cholesterol levels, and smoking. Proposed explanations for the increased risk include on-going HIV-replication in untreated individuals, immune suppression, HIV-associated inflammation, and the antiretroviral drugs [9-11].

HIV-infected individuals were recently reported to have higher circulating levels of IgG directed against copper-oxidized low-density lipoprotein (LDL) [12]. Oxidized forms of LDL (OxLDL) are major constituents of atherosclerotic plaques, the lesions causing CVD [13]. In plaques, OxLDL is taken up by macrophages, leading to foam cell formation. Antibodies directed against OxLDL may both exert pro- and anti-atherogenic effect, depending on the isotype, with IgG being mostly pro- and IgM chiefly anti-atherogenic [14]. Animal studies have demonstrated that mice lacking circulating IgM develop more atherosclerosis than mice with normal levels of IgM [15], and that immunization of mice with inactivated *Streptococcus pneumoniae*, which causes increased levels of IgM against modified LDL, is atheroprotective [16]. One mechanism by which natural antibodies of the IgM isotype may be protective against atherosclerosis is by inhibiting the uptake of OxLDL into macrophages.

In humans, natural antibodies are produced by IgM + memory-B-cells. HIV infection has been associated with a wide range of B-cell defects, including decreased B-cell survival [17], enhanced expression of markers of B-cell activation, reduction of subsets of memory B-cells, polyclonal hypergammaglobulinemia, and impaired antibody responses to immunizations, for example *S. pneumoniae*[18-25]. Our hypothesis was that HIV-infected (HIV+) individuals could have impaired production of natural antibodies that are protective against atherosclerosis in addition to their previously shown higher levels of IgG against OxLDL [12]. We therefore set out to determine if HIV + subjects, treatment naive or on ART, have disturbed levels of IgM and IgG antibodies directed against different forms of modified LDL as compared to uninfected subjects.

## Methods

### Study participants

Chronically HIV-1 (hereafter HIV) infected individuals were retrospectively included from the Department of Infectious Diseases, Sahlgrenska University Hospital, Gothenburg, Sweden between 2005 and 2010. Inclusion criterion was available stored plasma or serum sample. Exclusion criteria were a diagnosis of opportunistic infections, tumours, or co-infections with hepatitis B and/or C. HIV + subjects were divided into four different groups based on their immune status and treatment regimen. HIV + individuals who were treatment naive or who had been off ART for more than 6 months and had a CD4^+^ T-cell count < 250 × 10^6^/L constituted the first group. The second group was HIV + individuals who were treatment naive or who had been off ART for more than 6 months and had a CD4^+^ T-cell count > 500 × 10^6^/L. The other two groups consisted of HIV + individuals who had been on treatment for more than 12 months with a PI-based regimen or an NNRTI-based regimen, respectively. Lipid-lowering treatment (pravastatin) was used by two individuals on a PI-based regimen and by one individual treated with an NNRTI. Control plasma/serum samples were obtained from healthy HIV seronegative (HIV-) age- and gender matched blood donors during the same time period as the samples from the HIV + patients were collected. For samples matched to HIV + plasma samples, plasma was used for the analyses, and for samples matched to HIV + serum samples, serum was used. The following analyses were performed in all subjects: IgG and IgM directed against OxLDL and MDA-LDL, total IgM and IgG, highly sensitive C-reactive protein (CRP), apolipoprotein A1 (ApoA1), apolipoprotein B (ApoB), and soluble CD14 (sCD14). In HIV + subjects, plasma HIV RNA, blood CD4^+^ T-cell counts, serum cholesterol, HDL-cholesterol, and triglycerides were analyzed. The study was approved by the regional ethics committee in Gothenburg and informed consent was obtained from all study participants.

### Measurements of antibody levels

Levels of specific IgG and IgM directed against copper OxLDL and malondialdehyde-modified LDL (MDA-LDL) were determined in thawed plasma/serum from all study subjects by chemiluminescent enzyme-linked immunosorbent assay (ELISA) as previously described [26]. Briefly, microtiter plates were coated with OxLDL (5 mg/L) or MDA-LDL (5 mg/L) and the samples were added for one hour in dilutions of 1:200 (for IgG) or 1:100 (for IgM). OxLDL and MDA-LDL were prepared from LDL isolated from healthy male blood donors using the potassium bromide ultracentrifugation method as previously described [27,28]. Alkaline phosphatase-conjugated goat anti-human IgM (μ-chain specific, Sigma-Aldrich, Saint Louis, MO) and anti-human IgG (y-chain specific, Sigma-Aldrich) were used for detection of antibodies, and quantification was performed with Lumiphos 530 (Lumigen Inc., Southfield, MI) using LMaxII (Molecular Devices, Sunnyvale, CA). Parallel analysis of plasma and serum samples from 30 healthy individuals showed high concordance between results obtained in serum versus plasma: MDA-LDL IgG R^2^ = 0.96, OxLDL IgG R^2^ = 0.95, MDA-LDL IgM R^2^ = 0.98, OxLDL IgM R^2^ = 0.97. Thus, serum and plasma samples were analyzed together without factorization. In order to minimize the day to day and plate to plate variation, a calibrator (serum sample) was added to all microtiter plates, and RLU (relative luminescence unit) obtained for each sample was divided with the RLU obtained with the calibrator. In addition, a sample standard was included on all microtiter plates and a maximum deviation of ±2SD from the mean result of the standard was accepted. Using this approach, the inter- and intraassay CV for all specific antibodies measured was < 10%.

Total IgG and IgM levels were determined in thawed plasma/serum samples from all study subjects at the accredited local clinical chemistry laboratory with nephelometry using Immage 800 (Beckman Coulter, Brea, CA).

### Other laboratory analyses

ApoA1, ApoB, and CRP were determined with immunothurbidimetric methods (all from Roche Diagnostics Scandinavia AB, Bromma, Sweden) in thawed plasma/serum of all study individuals at the accredited local clinical chemistry laboratory. In HIV + individuals only, serum triglycerides, serum total-, LDL- and HDL-cholesterol were determined with immunothurbidimetric methods (all from Roche Diagnostics Scandinavia AB) in fresh serum samples at the time of entry into the study at the accredited local clinical chemistry laboratory. Plasma HIV RNA, and blood CD4^+^ T-cell counts were determined with Cobas Amplicor HIV-1 Monitor Test, version 1.5 (Roche AB, Basel, Switzerland) and flow cytometry at the accredited local clinical laboratories of virology and immunology, respectively. Circulating sCD14 levels were determined in plasma/serum samples of all study subjects using Quantikine Human sCD14 Immunoassay ELISA (R&D Systems, Minneapolis, MN).

### Statistical analyses

All statistical analyses were performed using SPSS Software package 19 (SPSS Inc., Chicago, IL). Log_10_ transformation was applied to all HIV RNA data. Data were analyzed with Mann–Whitney *U* test for comparisons between the HIV + patients and the HIV- controls, and with Kruskal-Wallis one-way analysis of variance followed by Dunn’s test for multiple comparisons. Relationships between continuous variables were analyzed using Spearman correlation. P <0.05 was considered statistically significant.

## Results

### Clinical characteristics of the study subjects

Ninety-one HIV + subjects were included in total and divided into four groups based on treatment regimen and immune status; (1) treatment naive or having been off ART > 6 months with CD4^+^ T-cell count < 250 × 10^6^/L (n = 22), (2) treatment naive or having been off ART > 6 months with CD4^+^ T-cell count > 500 × 10^6^/L (n = 22), (3) treatment for > 12 months with a PI-based regimen (n = 24), and (4) treatment for > 12 months with an NNRTI-based regimen (n = 23). The clinical characteristics of the HIV + individuals and the HIV- controls (n = 92) are summarized in Table 1. Treatment-naive HIV + subjects had a significantly shorter time from diagnosis to study entrance than HIV + subjects on treatment, particularly subjects with CD4^+^ T-cell count < 250 × 10^6^/L. Most of these individuals started ART shortly after being diagnosed with HIV. Among untreated subjects, all but one was naive to ART. The patient who previously had been treated with ART had been without treatment for almost five years. The two treated groups showed a comparable duration of therapy. All treated individuals had undetectable plasma HIV RNA levels (< 20 copies/mL).

**Table 1:** Demographic and treatment characteristics of study participants

	**Subject group**					
	**CD4 < 250**	**CD4 > 500**	**ART (PI/r)**	**ART (NNRTI)**	**HIV-**	**p-value**
	**n = 22**	**n = 22**	**n = 24**	**n = 23**	**n = 92**	**–**
Sex (male:female)	12:10	16:6	11:13	18:5	56:36	ns
Age in years	36 (22–61)	45 (20–73)	41 (21–65)	46 (27–62)	44 (20–73)	ns
Months since HIV-diagnosis	0 (0–101)	30 (3–207)	152 (32–305)	155 (50–302)	–	***
CD4 nadir (× 10^6^ cells/l)	170 (30–250)	480 (300–1000)	175 (0–310)	150 (0–470)	–	***
CD4 at baseline (× 10^6^ cells/l)	170 (40–250)	605 (520–1000)	565 (360–1100)	470 (270–900)	–	***
HIV RNA (log_10_ copies/ml)	4.89 (2.89–5.93)	3.87 (< 1.28–5.12)	All < 1.28	All < 1.28	–	***
Pre-ART HIV RNA (log_10_ copies/ml)	–	–	5.05 (2.63–6.62)	4.88 (2.92–5.79)	–	**
Treatment duration (months)	–	–	57 (28–204)	77 (28–210)	–	ns
Apolipoprotein B (g/L)	0.81 (0.47–1.40)	0.92 (0.47–1.40)	1.10 (0.33–1.50)	1.00 (0.47–1.80)	0.87 (0.42–2.20)	*
Apolipoprotein A1 (g/L)	1.40 (0.76–2.10)	1.45 (0.94–1.90)	1.55 (1.10–1.90)	1.70 (1.10–2.80)	1.50 (1.10–2.60)	ns
Total cholesterol (mmol/L)	4.2 (3.0–8.4)	5.0 (3.6–7.1)	5.7 (2.4–7.8)	6.0 (3.6–9.5)	–	***
HDL-cholesterol (mmol/L)	1.3 (0.6–2.1)	1.3 (0.6–2.1)	1.2 (0.9–2.0)	1.5 (0.9–2.7)	–	ns
Triglycerides (mmol/L)	1.20 (0.49–4.30)	1.45 (0.50–2.50)	2.15 (0.78–9.40)	1.70 (0.71–12.00)	–	**
hsCRP (mg/L)	2.75 (0.17–50.00)	1.60 (0.00–12.00)	1.40 (0.00–14.00)	3.50 (0.34–11.00)	0.63 (0.0–36.00)	***
Soluble CD14 (ng/mL)	1333 (991–3762)	1491 (842–1888)	1621 (1067–2283)	1796 (1228–3528)	1259 (872–2131)	***

Significance testing was performed for all five groups where data was available for HIV- controls, otherwise only for HIV + subjects. ART: antiretroviral treament; PI/r: ritonavir-boosted protease inhibitor; NNRTI: non-nucleoside reverse transcriptase inhibitor; hsCRP: highly sensitivity C-reactive protein. All values are given as median (range). NS: not significant; *p < 0.05; **p < 0.01; ***p < 0.001.

### Elevated IgG and reduced IgM levels directed against modified LDL in HIV + subjects

**Figure 1:**
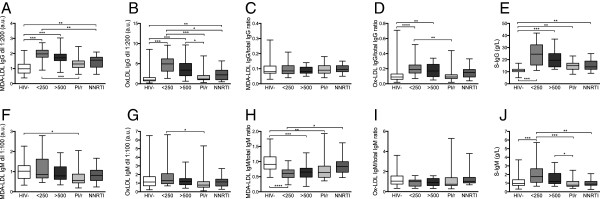
**Concentrations of total and oxidized forms of IgM and IgG in HIV- controls and the different HIV + groups.** Figure shows HIV-negative controls (HIV-), untreated HIV + subjects with CD4 < 250 (< 250) or > 500 (> 500), and HIV + treated subjects on a boosted protease inhibitor regimen (PI/r) or an NNRTI regimen (NNRTI). **A**. MDA-LDL IgG, **B**. OxLDL IgG, **C**. Ratio MDA-LDL IgG/total IgG, **D**. Ratio OxLDL IgG/total IgG, **E**. Total IgG, **F**. MDA-LDL IgM, **G**. OxLDL IgM, **H**. Ratio MDA-LDL IgM/total IgM, **I**. Ratio OxLDL IgM/total IgM, **J**. Total IgM. Boxes encompass interquartile ranges with median (line), whiskers designate the range. *p < 0.05, **p < 0.01, ***p < 0.001, a.u.: arbitrary units.

HIV + subjects had significantly higher levels of IgG directed against both MDA-LDL and OxLDL compared to HIV- controls (Figure 1A–B). The highest levels of OxLDL IgG were detected in untreated patients with CD4^+^ T-cell counts < 250 × 10^6^/L. When IgG directed against MDA-LDL and OxLDL were normalized to total IgG, expressed as the ratio of MDA-LDL IgG/total IgG, the increase in OxLDL IgG persisted although there were no significant differences in the ratio of MDA-LDL IgG/total IgG between HIV + individuals and uninfected controls (Figure 1C–D). Total IgG levels were significantly higher in all HIV + individuals compared to uninfected controls (Figure 1E). For IgM directed against MDA-LDL and OxLDL, the pattern differed from that found for IgG (Figure 1F–I). HIV + patients displayed lower levels of IgM directed against MDA-LDL, both when MDA-LDL IgM was analyzed alone and when it was normalized to total IgM, expressed as the ratio of MDA-LDL IgM/total IgM (Figure 1F–G). Multiple comparisons showed that all HIV + subjects, except those on treatment with an NNRTI-based regimen, had lower ratios of MDA-LDL IgM/total IgM levels compared to HIV- controls. Within the group of HIV + individuals, total IgM levels were highest in treatment naive patients with low CD4^+^ T cell counts (Figure 1J).

### Correlations between antibody levels and viral load

As expected, there were strong correlations between MDA-LDL and OxLDL IgG and MDA-LDL and OxLDL IgM, respectively (Table 2). Somewhat unexpected, there were strong correlations between total and MDA-LDL/OxLDL-specific IgGs and IgMs, with a consistent relationship between IgMs in both groups, but only in HIV + subjects for IgG (Table 2). IgG directed against MDA-LDL correlated with HIV RNA levels in individuals with detectable viral loads (untreated HIV + subjects) (r_S_ = 0.338, p = 0.02, n = 43). There were no correlations between plasma HIV RNA levels and any of the other antibodies (MDA-LDL IgM, OxLDL IgM and IgG, and total IgM and IgG) (data not shown). OxLDL IgG (r_s_ = 0.236, p < 0.05), total IgG (r_s_ = 0.284, p < 0.01), and total IgM ((r_s_ = 0.269, p < 0.05), but not the remaining antibodies, correlated with the CD4^+^ T-cell count in HIV + subjects.

**Table 2:** Correlations among HIV + participants

	**MDA-LDL IgG**	**OxLDL IgG**	**Total IgG**	**MDA-LDL IgM**	**OxLDL IgM**	**Total IgM**
**MDA-LDL IgG**	N/A	rs = 0.812	rs = 0.566	rs = 0.108	rs = 0.216	rs = 0.266
	N/A	p < 0.0001	p < 0.0001	p = 0.147	p = 0.003	p < 0.0001
**OxLDL IgG**	rs = 0.812	N/A	rs = 0.614	rs = 0.126	rs = 0.252	rs = 0.319
	p < 0.0001	N/A	p < 0.0001	p = 0.088	p = 0.001	p < 0.0001
**Total IgG**	rs = 0.566	rs = 0.614	N/A	rs = 0.038	rs = 0.120	rs = 0.391
	p < 0.0001	p > 0.0001	N/A	p = 0.612	p = 0.109	p < 0.0001
**MDA-LDL IgM**	rs = 0.108	rs = 0.126	rs = 0.038	N/A	rs = 0.842	rs = 0.707
	p = 0.147	p = 0.088	p = 0.612	N/A	p < 0.0001	p < 0.0001
**OxLDL IgM**	rs = 0.216	rs = 0.252	rs = 0.120	rs = 0.842	N/A	rs = 0.663
	p = 0.003	p = 0.001	p = 0.109	p < 0.0001	N/A	p < 0.0001
**Total IgM**	rs = 0.266	rs = 0.319	rs = 0.391	rs = 0.707	rs = 0.663	N/A
	p < 0.0001	p < 0.0001	p < 0.0001	p < 0.0001	p < 0.0001	N/A
**hsCRP**	rs = 0.231	rs = 0.193	rs = 0.286	rs = 0.035	rs = 0.063	rs = 0.060
	p = 0.002	p = 0.009	p < 0.0001	p = 0.63	p = 0.40	p = 0.43
**Soluble CD14**	rs = 0.097	rs = 0.195	rs = −0.348	rs = −0.199	rs = −0.177	rs = −0.326
	p = 0.358	p = 0.064	p = 0.001	p = 0.059	p = 0.093	p = 0.002

hs: higly sensitive C-reactive protein, rs: Spearman correlation coefficient.

### Treatment-related differences in serum cholesterol and triglycerides in HIV + subjects

**Figure 2:**
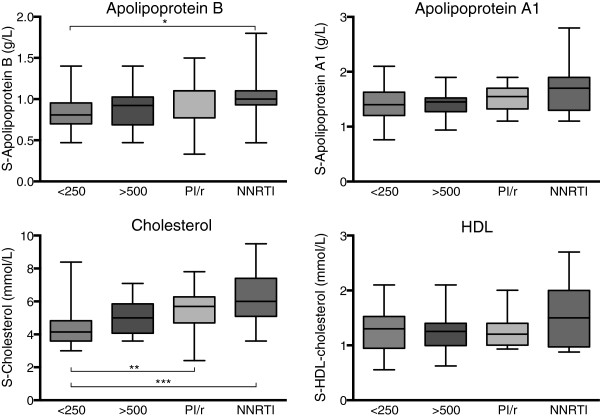
**Concentrations of apolipoproteins, total cholesterol and HDL cholesterol in the different HIV + groups.** Figure shows untreated HIV + subjects with CD4 < 250 (< 250) or > 500 (> 500), and HIV + treated subjects on a boosted protease inhibitor regimen (PI/r) or an NNRTI regimen (NNRTI). Boxes encompass interquartile ranges with median (line), whiskers designate the range. *p < 0.05, **p < 0.01, ***p < 0.001.

The pattern of increased levels of IgG directed against OxLDL and MDA-LDL and decreased levels of IgM directed against these epitopes has previously been associated with an increased risk of CVD. Since this association has not been shown to be independent of other risk factors for CVD, we assessed other markers for cardiovascular risk in our study individuals. None of the apolipoproteins ApoB and ApoA1 differed significantly between HIV + and uninfected individuals. In a multiple comparison test within the HIV + group, individuals on treatment with an NNRTI-based regimen had higher ApoB levels than treatment naive patients with CD4^+^ T-cell count < 250 × 10^6^/L (Figure 2 and Table 1). Serum cholesterol, which was determined only in the HIV + individuals, also differed significantly between the different HIV + groups (Figure 2 and Table 1). As expected, there were positive correlations between ApoB levels and cholesterol concentrations, as well as between ApoA1 and HDL-cholesterol (r_S_ = 0.812 and 0.814, respectively, both p < 0.001). In regard to serum triglyceride concentrations, patients on a PI/r based regimen had the highest serum triglyceride concentrations (Table 1).

### Inflammation markers

One explanation for increased levels of total IgG in HIV + individuals is a general activation of the immune system, either due to HIV per se or due to other microorganisms. One such mechanism described in individuals with HIV is microbial translocation, and we therefore analyzed sCD14 in addition to the inflammation marker also proposed as a marker of cardiovascular risk, CRP. Levels of sCD14 were higher in HIV + subjects as a group compared to HIV- controls (Table 1) with the highest levels in patients on an NNRTI-based regimen (Table 1). There were no correlations between CRP or sCD14 and plasma HIV RNA levels (data not shown). There was an inverse correlation between circulating levels of sCD14 and total IgG and IgM, but no relationship between sCD14 and specific IgG or IgM directed against OxLDL or MDA-LDL (Table 2), or HIV RNA levels (data not shown). On the other hand, there was an inverse correlation with levels of sCD14 and CD4^+^ T-cell count (r_s_ = −0.297, p < 0.01).

## Discussion

In this study, we show that chronic HIV infection is associated with elevated levels of IgG against oxidized forms of LDL in addition to elevated levels of total IgG. We also show that levels of these specific antibodies are related to plasma HIV RNA levels and treatment regimen, but only weakly with inflammatory status.

The main objective of this study was to determine if HIV + subjects have disturbed levels of IgG or IgM antibodies against oxidized forms of LDL compared to HIV- subjects. We found a decrease in the levels of potentially atheroprotective IgM directed against MDA-LDL and a major increase in the levels of IgG directed against both MDA-LDL and OxLDL. High levels of IgGs directed against oxidized forms of LDL, particularly in the form of immune complexes, have previously been associated with increased development of atherosclerosis and CVD [29,30]. In addition, anti-OxLDL-IgG might be consumed in the event of an erupted atherosclerotic plaque as indicated by low levels of anti-OxLDL IgG in acute coronary syndromes [31]. Low IgM levels against oxidized forms of LDL and similar epitopes have consistently been shown to be associated with an increased risk of atherosclerosis [32-34]. In mice, shifting the ratio IgG/IgM towards lower IgM and higher IgG was associated with aggravated atherosclerosis, indicating protection by IgM and harmfulness of IgG [35].

Da Cunha et al. recently reported higher IgG levels against OxLDL in HIV-infected individuals [12]. Our study confirms and further extends those observations. We show that HIV + patients have higher levels of IgG directed also against another form of modified LDL, MDA-LDL, as well as of total IgGs. These results implicate that anti-OxLDL IgG levels in HIV + individuals reflect a higher activity of the humoral immune system in these individuals. Regardless of the mechanism for the increase, the IgGs might play an important role for the risk of developing atherosclerosis, especially in combination with lower levels of IgM against modified LDL, resulting in a possibly adverse anti-OxLDL IgG/IgM ratio.

HIV infection leads to alterations in the lipid profile [36,37], and ART also has effects on triglyceride and cholesterol levels [38,39]. The incidence of dyslipidemia after the introduction of combination ART ranges between 15 and 30%, with hypertriglyceridemia being the most common change [40]. Among the HIV + subjects, treated patients receiving PI/r-based regimens had the highest triglyceride levels. Triglyceride concentrations were not analyzed in HIV- controls. HIV + individuals on ART had the highest levels of total cholesterol, and untreated subjects with low CD4^+^ T-cell counts, the lowest levels. It has previously been shown that serum lipid levels uniformly decline following HIV-seroconversion and after initiation of ART there is a rise in total cholesterol and LDL that typically exceeds pre-infection levels, whereas the recovery of HDL may be incomplete [36]. In HIV + individuals, the concentration and size of LDL might be of limited value for the risk of CVD [41]. Other potential markers of cardiovascular risk are ApoB and ApoA1, the signature proteins of LDL and HDL particles, respectively. In this study, there were no significant differences in apolipoproteins between HIV + individuals and HIV- subjects. Although there were correlations between levels of ApoB and cholesterol, the differences between groups were more pronounced for cholesterol than for ApoB.

As expected, HIV + subjects in our study had higher hsCRP levels than HIV- controls. The reason for HIV-associated immune activation is not fully understood, but is likely to be multifactorial. Important factors include direct effects of the virus and/or viral proteins, co-infections with other pathogens, innate and adaptive immune responses to HIV, persistent elevation of type I and II interferons, non-antigen specific bystander activation of immune cells, dysregulated cytokine and chemokine production, and bacterial products that translocate from the gut [42-44]. Immune activation is not only present in untreated HIV + individuals, but also in treated suppressed patients. As is the case for hsCRP, sCD14 levels do not seem to normalize despite several years of effective combination ART [45]. In this study, we found higher levels of sCD14 in HIV + individuals than in HIV- controls. There was a correlation between sCD14 and CRP, but no correlation between sCD14 and antibodies against oxidized forms of LDL. Since there was an inverse relationship with total IgG and IgM and no relationship between sCD14 and specific anti-OxLDL antibodies, it is unlikely that the source of these antibodies is an increased bacterial load reflecting for example bacterial translocation.

This study was designed to detect differences in levels of antibodies directed against oxidation-specific epitopes. However, the sizes of the subgroups were rather small, resulting in limited power to detect differences in other markers assessed in the study and to identify associations between different parameters.

## Conclusions

In conclusion, HIV infection is associated with higher levels of IgG including specific IgG against oxidized forms of LDL, and lower IgM against the same epitope. In addition to dyslipidemia, immune activation, HIV-replication and an accumulation of risk factors for atherosclerosis risk factors, this adverse antibody profile may be of major importance for the increased risk of CVD in HIV-infected individuals.

## Competing interests

None of the authors have any conflicts of interest.

## Authors’ contributions

LF conceived of the study. AY and LF designed the study, analyzed the data and performed statistical analysis. AY drafted the manuscript and included subjects for the study. LF and KJ performed all analysis. All authors read and approved the final manuscript.

## Pre-publication history

The pre-publication history for this paper can be accessed here:

http://www.biomedcentral.com/1471-2334/14/143/prepub
